# Mast cell mediators cause the early allergic bronchoconstriction in guinea pigs in vivo: a model of relevance to asthma

**DOI:** 10.1186/1476-9255-10-S1-P18

**Published:** 2013-08-14

**Authors:** Jason P Riley, Barbara Fuchs, Lisa Sjöberg, Gunnar P Nilsson, Lars Karlsson, Sven-Erik Dahlén, Navin L Rao, Mikael Adner

**Affiliations:** 1 JanssenResearch & Development, LLC, 3210 Merryfield Row, San Diego, CA 92121, USA; 2 Experimental Asthma and Allergy Research, Institute of Environmental Medicine, Karolinska Institutet, SE-171 77 Stockholm, Sweden; 3 Clinical Immunology and Allergy Unit, Department of Medicine, Karolinska Institutet, SE-171 77 Stockholm, Sweden

## Background

One feature of allergic asthma, the early allergic reaction (EAR), is not present in the commonly used mouse models. We therefore investigated the mediators involved in EAR in a guinea pig *in vivo* model of allergic airway inflammation.

## Method

Animals were sensitized using a single ovalbumin (OVA)/alum injection and challenged with aerosolized OVA on day 14. On day 15, airway resistance was assessed after challenge with OVA or methacholine using the forced oscillation technique, and lung tissue was prepared for histology.

## Results

The contribution of mast cell mediators was investigated using histamine 1 receptor (pyrilamine) and cysteinyl leukotriene 1 receptor (montelukast) antagonists, and the cyclooxygenase inhibitor indomethacin as single treatment, or as dual or triple combinations. OVA-sensitized and challenged animals demonstrated airway hyperresponsiveness to methacholine, and lung tissue eosinophilic inflammation. Antigen challenge induced a strong EAR in the sensitized animals. Treatment with a single compound, or indomethacin together with pyrilamine or montelukast, did not reduce the antigen-induced airway resistance. In contrast, dual treatment with pyrilamine together with montelukast, or triple inhibitor treatment, attenuated approximately 70% of the EAR.

## Conclusion

We conclude that, as in humans, the guinea pig allergic inflammation model exhibit both EAR and airway hyperresponsiveness, supporting its suitability for *in vivo* investigations of the effects of mast cell activation with relevance to asthma. Moreover, the known mast cell mediators histamine and leukotriene caused the main part of the EAR. The data lend further support to the concept that combination therapy with selective inhibitors of key mediators could improve asthma management.

